# Evaluation of a Pilot Medical Student-Resident Liaison Program in Psychiatry

**DOI:** 10.1007/s40670-025-02404-w

**Published:** 2025-05-05

**Authors:** Sezai Ustun Aydin, Ozlem Bozdagi Gunal, Efe Sari, Daniella Colombo, Lisette Nazario, Petros Levounis

**Affiliations:** 1 Department of Psychiatry, Child & Adolescent Psychiatry, Rutgers Robert Wood Johnson Medical School, New Brunswick, NJ USA; 2 Department of Psychiatry, Rutgers New Jersey Medical School, Newark, NJ USA; 3 School of Medicine, Acibadem University, Istanbul, Turkey; 4 Department of Psychiatry, Boston Medical Center, Boston, MA USA

**Keywords:** Medical education, Medical student resident liaison, Mentorship, Qualitative, Quantitative, Focus group discussion

## Abstract

**Objective:**

As psychiatry residency programs grow increasingly competitive, innovative mentorship models are needed to support medical students’ academic development. This study evaluates the Medical Student Resident Liaison (MSRL) Program, implemented in the academic year 2023–2024, aimed at connecting medical students to psychiatry-related scholarly opportunities, including research, teaching, and networking.

**Methods:**

Eighteen participants completed surveys assessing changes in knowledge, interest, project access, and confidence in their residency applications. Additionally, a focus group interview with medical students provided qualitative insights, analyzed through thematic analysis to identify key experiences and areas for improvement.

**Results:**

Survey analysis revealed significant increases in students’ perceived knowledge (*p*=0.001), access to psychiatry projects (*p*=0.004), and confidence in their residency application CVs (*p*=0.039). The focus group identified key benefits of the program, such as enhanced access to research opportunities, networking, and autonomy in project involvement.

**Conclusion:**

The MSRL program effectively enhanced students’ academic growth, confidence, and engagement with psychiatry. It serves as an adaptable model for addressing limited faculty availability and increasing interest in psychiatry residency. Implementing structured mentorship programs as early as the first or second year of medical school may further enhance student preparedness, particularly if coupled with faculty-led networking opportunities. As previous research indicates, residents’ unique position in mentorship is associated with better medical student outcomes, and we advise the implementation of the MSRL program to all interested specialties.

**Supplementary Information:**

The online version contains supplementary material available at 10.1007/s40670-025-02404-w.

## Introduction

Following the transition of the USMLE Step 1 to a pass/fail system, medical students sought to distinguish their residency application by participating in more projects related to their specialty of interest [1–3]. In the 2024 National Residency Matching Program (NRMP) Match, US MD students who successfully matched to psychiatry had an average of 7.5 abstracts, presentations, and publications, compared to 4.6 for those who did not match [4]. Medical students are also increasingly eager to pursue careers in psychiatry [5]. As NRMP data show rising competitiveness in psychiatry, medical students may now face greater challenges in securing residency positions in the field [6]. The barrier to medical students accessing research, teaching, or other academic activities is not always due to a lack of scholarly opportunities, but often stems from a shortage of research mentors who can assist students [7, 8]. Mentorship in medicine is defined as “a dynamic, context-dependent, goal-sensitive, mutually beneficial relationship between an experienced clinician and junior clinicians and/or undergraduates focused upon advancing the development of the mentee.” Unlike supervising or role-modeling, mentorship may require departmental effort to establish [9]. Altogether, this renewed understanding of medical education highlights the need for innovative mentorship models that support medical students in their academic development [6].

Clinical leadership skills are highly valued due to their association with improved patient care and outcomes. Improving leadership skills is critical for better outcomes for both patients and health systems [10]. The Accreditation Council for Graduate Medical Education, a graduate medical education (GME)-accrediting body in the USA, has emphasized the need for leadership development during residency training [11]. Moreover, residents’ role as teachers has been increasingly emphasized in graduate medical education [12]. Near‐peer mentorship—where senior trainees mentor junior trainees—has been shown to foster quicker rapport, promote a safe, comfortable educational environment, and encourage the open exchange of ideas [13, 14]. Near-peer mentors also play a crucial role in preventing drop-offs in mentorship programs and promoting flexible, sustainable mentee-mentor relationships. Ultimately, mentees “pay forward” the mentorship they receive, thereby further boosting recruitment into psychiatry [15]. Also, Lopez et al. note that having a resident mentor is associated with statistically significantly increased odds of matching, likely due to residents’ unique ability to introduce and advocate for mentees to their faculty [16].

Given the increasing need for psychiatry-related scholarly opportunities and mentorship in medical education, structured initiatives like the Medical Student Resident Liaison (MSRL) program may help address this gap. In this model, residents work with faculty to support medical students’ interest and engagement in psychiatry through activities such as research, teaching, community involvement, and interest group mentoring. Although psychiatry interest groups in medical schools promote community engagement, they often focus on student-led experiences and lack formalized roles for both students and faculty [17]. They may also encounter challenges due to differing beliefs between students and faculty on what to support and promote [18]. The Department of Psychiatry at Rutgers New Jersey Medical School launched the MSRL program in the academic year 2023–2024 with the aim of connecting medical students with psychiatry-related scholarly opportunities, including research, publications, advocacy, and teaching other medical students under the supervision of a faculty. To carry this out, a designated resident regularly collected updates about new and ongoing departmental activities and shared them with students via email. The resident also met with students, student groups, and faculty to identify and facilitate opportunities. The medical students who expressed interest were matched with projects and supervisors based on their preferences, giving them the flexibility to participate at their own pace. Research indicates that students have a more positive experience when they work with mentors they choose rather than being assigned [19]. This structure provided medical students with ongoing support and opportunities to participate in various activities while giving faculty and other residents a consistent point of contact for student involvement in projects. Additionally, the program organized meetings with the faculty members to help students explore various psychiatry career paths.

Although there are examples of separate medical student or resident liaison programs, these programs do not specifically connect medical students with residents but rather with medical organizations [20, 21]. Bod et al. described a successful implantation of the MSR Liaison Program in Emergency Medicine and recommended implementing a similar program for different institutions and specialties [22]. Beyond these examples, Farkas et al. found that nearly all mentorship programs in medicine utilized either the traditional dyad model or a combination of dyad and group mentorship [6]. In their systematic review of 28 programs, 16 employed dyad mentorship, four used a group model, and seven implemented a combination of both. Most programs assigned faculty mentor(s) to the resident(s) or medical student(s), while some paired resident(s) or senior medical student(s) with the medical student(s). None utilized a strategy where residents could serve as mentors and connectors. Additionally, these programs commonly faced barriers such as costs and limited faculty availability, which Farkas et al. suggest could be addressed by adopting a student-as-mentor approach [6]. However, it is still important to remember that the mentor-mentee relationship is highly context-specific and organization-dependent, making comparisons between programs challenging, meaning an approach that works well in one context or organization may not be suitable in another [23].

We hypothesized that the MSRL program would stimulate interest in psychiatry among medical students, increase access to projects, enhance knowledge of the field, and improve confidence in students’ residency applications. Furthermore, we designed a focus group interview with a subsample of students for their detailed feedback regarding the MSRL program.

## Method

### Study Design

The Rutgers New Jersey Medical School Institutional Review Board approved this study (Pro2023002527). All medical students whom the MSRL program connected to activities were voluntarily recruited for this study. The activities included posters, publications, teaching, research, and meetings with faculty in the academic year of 2023–2024. The MSRL program ran from July 2023 to June 2024. The survey took place at the end of the academic year, and the focus group took place during July 2024. This program was open to all students, regardless of consenting to participate in this study or not. The M4 students in this study represent a group of medical students who were rising third-years when they started the program. Thus, almost all participants had the opportunity to discuss their research contributions during their residency applications. All the participating students (*n*=25) received an anonymous emailed link to fill out an online survey via Qualtrics XM [24]. The survey (Online Resource 1) included items such as *age, race/ethnicity, gender, medical school year, and type of project(s).* We also asked the students to rate *changes in their knowledge, interest, project access, and confidence in their CV for application after participating in the MSRL program* on a 5-point Likert scale. Finally, we asked the students for their opinions on *whether the MSRL program should be included in other departments or institutions*. Some medical students were involved in multiple projects, but each participant completed one survey regardless of the number of projects. All participants received three emails—the first inviting them to complete the survey and the subsequent two with reminders to do so. The reminders were sent in the 2 weeks following the initial invitation to complete the voluntary survey. Twenty-three medical students responded to the survey invitation. Seven students were removed because they did not consent (*n*=2) or provided incomplete answers (*n*=5). Eighteen consenting survey responders were included in the study (72%).

**Fig. 1:**
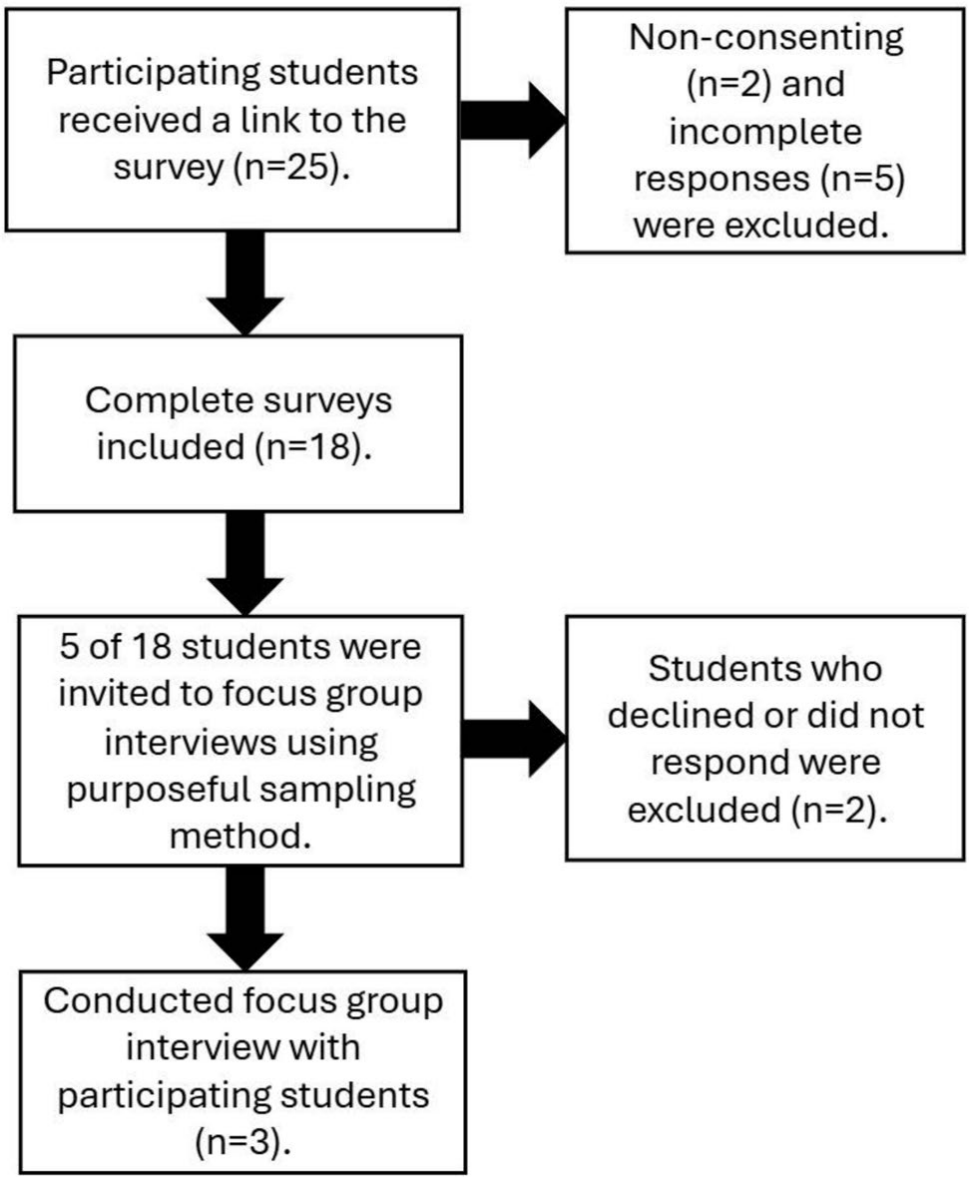
The inclusion process of the participants

We also obtained qualitative data by conducting a focus group interview. Through a purposeful sampling method, five of the medical students who participated in MSRL projects were invited via email to participate in this focus group interview to explore their experiences with the MSRL program. Their email addresses were available, as it was the primary mode of communication for MSRL project coordination. Although the invitation was extended to second-, third-, and fourth-year medical students, three fourth-year medical students interested in psychiatry residency agreed to participate in the interview (Figure 1). Although the sample size was relatively small, previous qualitative studies in medical education have shown that meaningful insights can emerge even with small cohorts of engaged participants [25].

The focus group was designed following a grounded theory approach, allowing medical students to share their direct opinions and experiences with the MSRL program [26, 27]. The focus group methodology was mainly selected to utilize the dynamic interaction arising from the differences and similarities in participants’ opinions during the interview, thereby enriching the depth and breadth of the conversation [28, 29]. An interview guide was developed based on results from a prior quantitative survey analysis, enhancing the validity of the discussion [30]. The interview guide and focus group questions are available in the supplementary material (Online Resource 2). During the focus group, one researcher moderated the session using the interview guide to facilitate discussion among participants. The focus group was conducted via an online meeting platform and, with informed consent, the meeting was recorded and the transcript later anonymized. Participants were compensated with a gift card for their time.

### Quantitative Statistics

Each pair of variables and outcome measures was compared via the Wilcoxon signed-rank test with continuity correction. Statistical significance was set at *p* < 0.05. Statistical analysis was carried out via R version 4.4.0 [31].

### Qualitative Analytic Plan

The primary objective of the focus group was to gain a deeper understanding of students’ experiences in the MSRL program and assess its impact on their perceptions of their academic careers. To identify patterns in the common experiences of medical students, we employed thematic analysis with an inductive approach. This method did not attempt to fit the data into any preexisting theories, allowing for flexibility and ensuring that the analysis was driven by the data itself, thereby capturing the authentic experiences of the participants [25, 33]. With this approach, we systematically and collaboratively coded the transcripts, grouped the codes into themes, and continuously refined the themes and the codes. This process ensured that our findings accurately represented the authenticity and nuances of the dataset, providing comprehensive insight into the participants’ experiences.

### Qualitative Coding Process

The transcripts were analyzed via step-by-step reflexive thematic analysis with an inductive approach described by Braun and Clarke [25]. First, ES and OG read the transcript multiple times to familiarize themselves with the dataset, taking notes on potential initial codes during this process. Second, units of text relevant to the research question and representing patterned responses with initial codes were labeled by ES. Some text segments were labeled with multiple codes based on their relevance to the research question, resulting in 51 initial codes. Third, all initial codes were reviewed with OG to ensure consistency and accurately represent the entire dataset. Any disagreements between the coders were discussed multiple times and resolved through consensus. This process led to the refinement of our initial codes into final codes. After a second exhaustive review by ES and OG, the inductive thematic analysis yielded seven final codes and three overarching themes, representing the entire dataset concerning the research questions.

## Results

### Quantitative Results

Respondent students came from diverse backgrounds and different years of medical training. The sociodemographic characteristics of the 18 participants who consented to the study are presented in Table 1.

**Table 1:** Demographics of the participants

	***Mean ± SD***
Age	26.91± 3.36
Medical school year	***N***
1 st	2
2nd	1
3rd	5
4 th	10
Race/ethnicity	***N***
Asian	11
Hispanic	3
Black	2
White	2
Gender	***N***
Female	12
Male	6

Research activities had the highest number of student participants (*n*=10, 43%), followed by teaching activities (*n*=8, 35%) and meetings with faculty (*n*=5, 22%). Five students (22.7%) participated in multiple activities: three were involved in research and meetings, one in research and teaching, and one in teaching and meetings. Notably, six participants (*n*=6, 33%) indicated they were already committed to non-psychiatry residencies, all of whom participated in teaching activities.

**Fig. 2:**
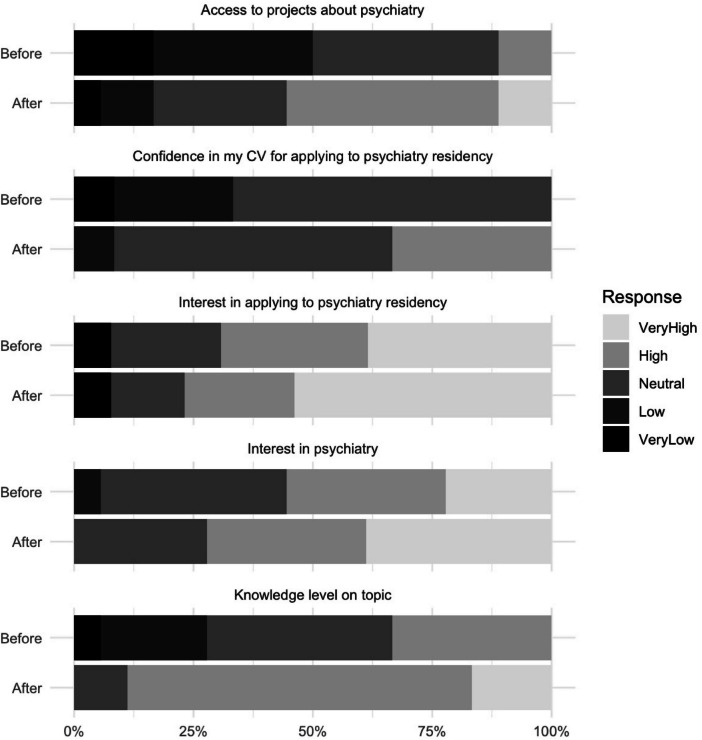
Survey responses before and after participation in the MSRL program

**Fig. 3:**
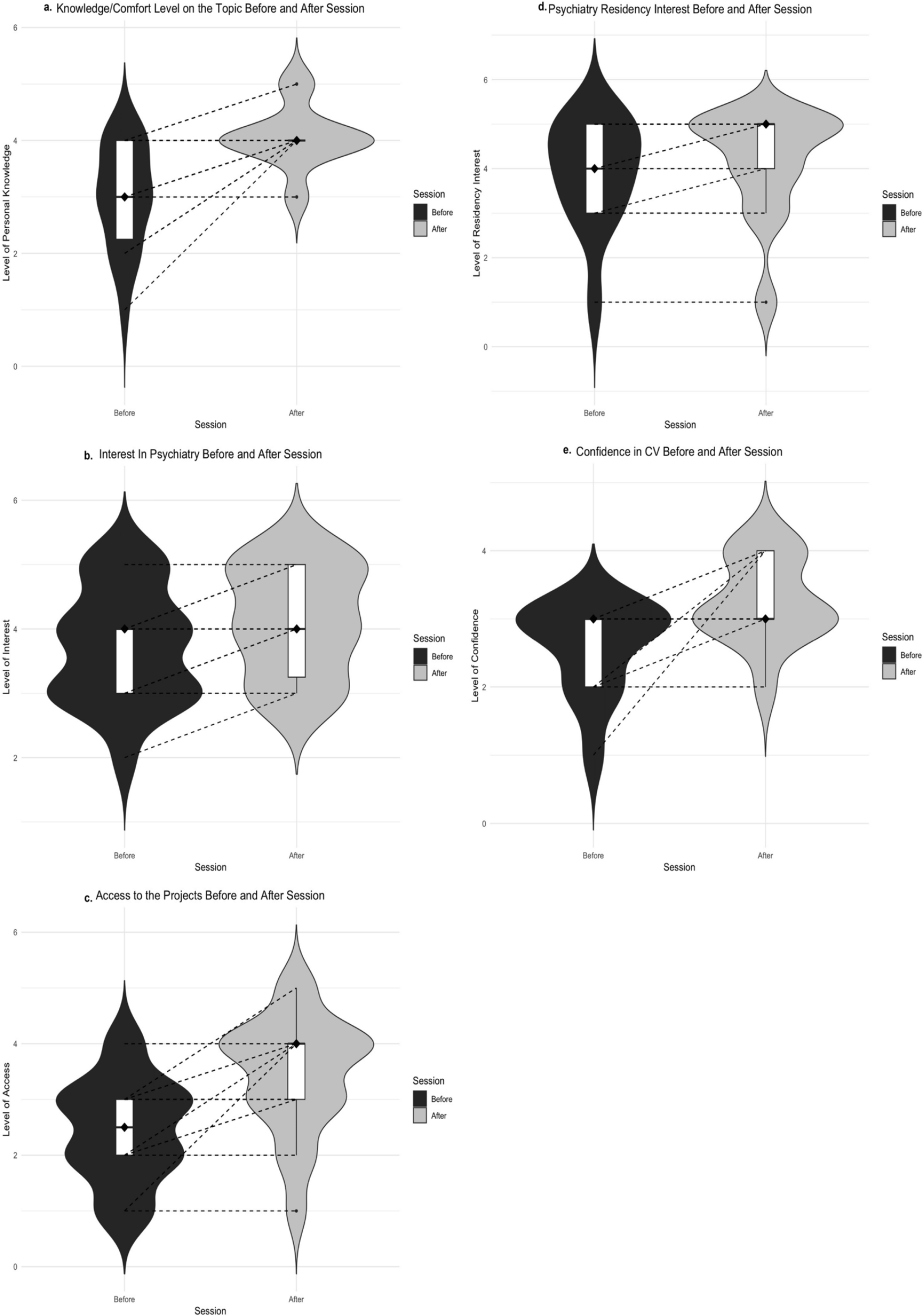
Perceived changes in medical students based on the survey answers. **a** Changes in students’ perceived knowledge or comfort levels before and after participating in MSRL activities (*p*-value = 0.001). **b** Changes in students’ interest in psychiatry before and after participating in MSRL activities (*p*-value = 0.008). **c** Changes in students’ access to psychiatry projects before and after the MSRL program (*p*=0.004). **d** Changes in students’ interest in applying for psychiatry residency before and after MSRL activities (*p* = 0.083). **e** Changes in students’ confidence in their CV for applying to psychiatry before and after MSRL activities (*p*=0.039)

The students’ answers were collected using a Likert scale (Figure 2). Students were asked to compare their knowledge and comfort levels before and after participating in an MSRL activity. The students reported a statistically significant increase in their perceived knowledge or comfort levels post-session (*p* = 0.001). The students were subsequently asked how the sessions affected their interest in psychiatry. All reported either increased interest or no change. The Wilcoxon signed-rank test showed a statistically significant increase in interest post-session (*p* = 0.008). When asked about their access to psychiatry-related projects, all students reported either increased access or no change, with the analysis showing a statistically significant increase (*p* = 0.004). Regarding interest in applying to a psychiatry residency program, most students already had high or very high interest pre-session, and none reported a decrease afterwards. There was no statistically significant change in interest in applying for psychiatry residency (*p* = 0.083). Finally, students were asked about their confidence in their CVs to apply to psychiatry residency. None of the students reported a decrease in their confidence. A statistically significant increase in confidence in their CVs was observed post-session (*p* = 0.039) (Figure 3). This suggests that structured mentorship not only facilitates academic involvement but may also play a critical role in students’ self-perception of preparedness for residency applications. An average of eight medical students per year participated in research within the psychiatry department during the academic years 2020–2023, before the MSRL program (SD=4.54). Following the introduction of the MSRL program, in the academic year 2023–2024, this number increased by 62.5%, to 13 students, who participated in basic and clinical psychiatry research.

### Qualitative Results

The primary themes of the focus group were “Academic advancement and growth” and “Areas for improvement and consideration.” The full codebook is included in Table 2.

**Table 2:** Codebook

**Theme**	**Description**	**Secondary codes**
Academic advancement and growth	Aspects of the program that would promote academic career growth for the majority of medical students	Research opportunity
		Learning opportunity
		Network opportunity
		Decision making
Areas for improvement and consideration	Aspects of the program that students identified as challenging and recognized as drawbacks for the majority of participants	Email format
		Regretting the project signed up
		Importance of early exposure

#### Theme 1: Academic Advancement and Growth

The participants agreed that the MSRL program was beneficial for enhancing their academic careers. During the semi-structured interview, the moderator asked questions to explore further the factors contributing to the participants’ positive experiences. When asked about their initial motivation for joining the program, students consistently emphasized three key opportunities: research, learning, and networking.

I don't really have any connections. So, it's not easy for a med student to go up to the attending and ask, ‘Hey, do you have any research projects?’ So, having this emailed out was much easier to get involved in.

When students were asked to reflect on their overall experience, the majority considered research opportunities to be the most beneficial aspect.

I think the research was more interesting or significant because I had a deeper involvement.

Research was a central focus of their discussions. For some, unfamiliarity with research and the scientific method posed challenges. One student expressed disappointment with standard aspects of the research process. Overall, we believe their research experience was valuable despite this, as they gained a deeper understanding of research methods and the time they demand. This participant mentioned:

It was just a matter of gathering enough people or samples to then translate that to literature. It was not like the discovery of a new thing; it was more like putting it out in the literature.

The other two students shared that they gained new insights and found the experience meaningful. Despite facing challenges typical of research methodology, they highlighted the following:

I was already interested, but I did learn about a new field aspect of psychiatry that I didn't know about before.

I guess it helped me become more creative in terms of my own ideas. Think it learning about like ‘how good of a case this is or how useful this research would be in the literature’.

The same two students also shared that their experience working on a research project with a resident differed from their previous research experiences, as they were actively involved in decision-making and felt their autonomy was respected.

Most of the research people end up doing is basically someone a year above them saying,'How about you do this project, you do all the dirty work, and then you put my name as first author.'Working with residents felt very different because they don’t need the research as much as we do, so they’re perfectly okay with us taking the lead and shaping the project.

#### Theme 2: Areas for Improvement and Consideration

During the interviews, the students identified several aspects of the MSRL program as areas for improvement. Two frequently mentioned issues were the email method of communication and the timing of research opportunity announcements.

While program coordinators chose email for its accessibility and ease of use, students expected more formal and detailed communication. This led many students to be unaware of the full scope of the program’s opportunities. Additionally, missed emails caused some students to overlook events and opportunities of interest.

I only did the research and not the lunch, and the reason is because of the fact that we're emailed about it, but I don't check my email that often, I think I have just really missed it.

Students reported regret for committing to early-announced projects when more appealing projects were shared later on. Students attributed this to the irregular timing of emails. They suggested that announcing all opportunities in a single, comprehensive communication would allow them to prioritize projects based on their interests and make more informed decisions.

If you find something interesting, you might sign up for it, but later on, you hear about something more interesting, but you're already committed. If there was a way to distribute or rank projects in order of preference, that would be helpful.

During the focus group, some students felt the MSRL program would have been more beneficial if introduced in the first or second year of medical school rather than the final year. They believed earlier exposure would have helped them build connections and engage in learning activities sooner, making them more equipped and confident.

I didn't know I wanted to do psych until my third year, and I feel if I had that experience back at MS1, MS2, maybe I would've seen how cool the field was.

However, one student expressed uncertainty about the benefits of early exposure for all medical students, noting that time commitments vary significantly among individuals.

I think it's helpful for everyone. If you're going into psych and you're interested in doing research at any point, you might have a lot of time given that your rotation or section is easier. I don't think it's particularly more beneficial to a certain year.

## Discussion

To the best of our knowledge, this is the first study that incorporated quantitative data from medical students’ surveys and qualitative data from a designed focus group interview to understand medical students’ experiences with MSRL psychiatry programs.

In line with the importance of introducing evidence-based research in medical education for better clinical outcomes [33], the MSRL program led to increased medical student participation in research within our department. Along with the increase in the number of students participating in research, they also reported greater knowledge and interest in psychiatry. Similar improvements were observed in students who participated in meetings with faculty and/or teaching activities. These findings are consistent with prior research from both the US and international contexts, demonstrating that structured mentorship enhances student engagement in academic projects [33].

The interest in psychiatry residency is increasing among medical students [33]. The MSRL program may help to expand access to psychiatry projects and strengthen students’ CVs for psychiatry residency applications. Moreover, the students who engaged in psychiatric enrichment activities during their medical education had the opportunity to gain valuable experience in interacting with patients and assuming clinical responsibility, which positively shaped their decision to pursue a career in psychiatry [33].

A significant portion of our participating students (33%) were already committed to non-psychiatry residencies, suggesting that the MSRL program appeals to a broader audience. As highlighted in the focus group, the ease of access to scholarly activities afforded by the program, described by one interviewee as “a good way to get your foot in the door,” may have contributed to this broader engagement. While the statistical analysis on the likelihood of applying for a psychiatry residency did not reach significance (*p* = 0.083), this might be due to the small sample size and high baseline interest in psychiatry residency among participants. A larger sample of students with more neutral or less favorable attitudes toward psychiatry would likely provide more accurate insights into attitude shifts. None of the students reported a decreased interest in applying to a psychiatry residency. In fact, one student shared during the focus group discussion that their participation in the MSRL program inspired them to pursue psychiatry residency—a reflection of the program’s potential impact. Given that nearly 45% of the US population experiences a shortage of mental health professionals [33], MSRL programs may help address this gap. To better attract medical students who are not initially committed to psychiatry and help them explore their interest earlier, Kishore et al. recommend strategies such as continuing mentorship programs within adult psychiatry residency training, which the MSRL program provides, as well as emphasizing residency applications and hosting patient-centered events [15].

Studies on residents’ and medical students’ perspectives on research during training have documented ambivalent attitudes toward its value [8]. However, another noteworthy finding of our study was that the participating medical students were highly engaged in research activities. This finding is consistent with other literature showing that many medical students view participation in research as necessary. They speculated that research adds weight to the curriculum vitae, notably when medical students believe their desired specialty to be competitive to get into [33]. Early exposure to research has also been linked to more positive attitudes toward research later in one’s career [8]. This type of early exposure, facilitated by the MSRL program, can help foster more favorable attitudes toward research. However, more research is needed, given that Kraft et al. suggested specialty-specific programs may be less beneficial for students who lack a foundational knowledge base in the field [33]. Also, it is worth acknowledging potential logistical challenges with implementing the MSRL program early in medical education, such as limited faculty availability and funding restrictions. Possible solutions may include integrating group-based mentorship models and seeking departmental support to cover the costs.

The focus group results contribute to the growing body of literature highlighting the importance of faculty support in medical students and emphasize how the absence of such programs can disadvantage their career development [33, 33]. Overall, the students expressed strong support for the MSRL program and endorsed efforts to enhance it for the benefit of future medical students. The recurring mention of research and networking opportunities as beneficial reflects their status as fourth-year medical students preparing to apply for residency. Given that a greater number of research experiences and outputs are associated with better match outcomes, the positive and negative experiences of our focus group may revolve around the research and networking opportunities provided by the MSRL program [33]. Also, previous literature highlights that mentor-mentee relationships can encounter challenges due to hierarchical dynamics, with issues arising when mentees become overly dependent on mentors or when mentors take on a paternalistic role, impeding effective mentoring and diminishing mentee autonomy [9]. The MSRL program, in contrast, creates a collaborative and supportive space where medical students take active roles in scholarly activities and decision-making. This approach was recognized during the focus group as a strength of working with residents.

## Limitations

This study had a high response rate of 72%, but the small size remains a major limitation of this study and should be considered when interpreting the results. However, a relatively small sample size allowed us to perform a focus group interview. Increasing participation in future iterations may be achieved by using multiple communication methods to increase the number of students participating in the program.

The responder bias and selection bias may be another concern due to the nature of the study. Although most participants were fourth-year students, those motivated to join the focus group may have been the ones who benefited the most from the program. Also, those who attended the MSRL programs may have been the students who were already highly motivated. Nonetheless, no student from any grade reported a dissatisfying experience in the surveys. However, we acknowledge that the retrospective pre/post design of the survey may have led students to unintentionally downplay their baseline levels of confidence, knowledge, or interest, especially knowing that the program was designed to improve these parameters. This form of participant bias may partly explain the absence of any reported decline in survey responses.

Another limitation we encountered while interpreting the results was the potential for recall bias among participants. However, no student reported a decline in any evaluated parameters before and after the MSRL program, suggesting the program’s benefit for all students.

Nonetheless, relying solely on quantitative data may still introduce recall bias. Therefore, this manuscript should be considered holistically, incorporating thematic analysis, which was semi-structured by the researchers to address some of the limitations of the quantitative results. Furthermore, we had no students participating in advocacy, partly due to a lack of ongoing advocacy projects. Another area that can be improved is that students reported a need for more frequent communication, such as at least quarterly updates from the program, regardless of whether they expressed any interest in psychiatry.

This study also compared the average number of students involved in psychiatry research to that in previous years and found an increase in the number of students participating in psychiatry research after implementing the MSRL program. However, we did not check for other possible confounding factors, so it is not possible to rule out a coincidental change. For future directions, we strongly advise similar programs to conduct longitudinal studies to explore the sustainability of the MSRL program and its impact on academic and match outcomes.

## Supplementary Information

Below is the link to the electronic supplementary material.

Supplementary file1 (PDF 176 KB)

Supplementary file2 (PDF 219 KB)

## Data Availability

The authors confirm that the data supporting the findings of this study are available within the article and its Supplementary material. Raw data supporting this study's findings are available from the corresponding author upon reasonable request.
