# Supplementary material for: Evaluation of a Pilot Medical Student-Resident Liaison Program in Psychiatry
Source: Med Sci Educ. 2025 May 5;35(4):2021–31. doi: 10.1007/s40670-025-02404-w (PMC12532957; doi:10.1007/s40670-025-02404-w)
Supplement: Supplementary file 1 — Supplementary file1 (PDF 176 KB) [file 40670_2025_2404_MOESM1_ESM.pdf]

Medical Student Resident Liaison Program Feedback

Start of Block: Block 1

This survey will be used to improve the Medical Student Resident (MSR) Liaison Program to help you better next year. Possibly, a publication with the results obtained from this survey will guide us implementing this program further. It consists of 13 questions. Please click here for the Consent form

- ○ I consent
- ○ I do not consent

Age:

____

Race / ethnicity

____

Gender

____

Medical School Year

____

End of Block: Block 1

Start of Block: Default Question Block

1 Type of participated projects (you can choose multiple):

- ▢ Research/Poster
- ▢ Teaching
- ▢ Lunch with Attendings
- ▢ Advocacy
- ▢ Other ____

---

2 Knowledge / comfort level on the topic BEFORE attending this project or session:

- ○ Very Low
- ○ Low
- ○ Neutral / Fair
- ○ High
- ○ Very High

---

3 AFTER attending this project or session:

- ○ Very Low
- ○ Low
- ○ Neutral / Fair
- ○ High
- ○ Very High

4 Interest in the psychiatry BEFORE attending this project or session:

- Very Low
- Low
- Neutral / Fair
- High
- Very High

5 AFTER attending this project or session:

- Very Low
- Low
- Neutral / Fair
- High
- Very High

6 Access to the projects about psychiatry BEFORE the MSR liaison program:

- Very Low
- Low
- Neutral / Fair
- High
- Very High

7 AFTER the MSR liaison program:

- ○ Very Low
- ○ Low
- ○ Neutral / Fair
- ○ High
- ○ Very High

---

8 My interest in applying to a psychiatry residency BEFORE attending this project or session:

- ○ Very Low
- ○ Low
- ○ Neutral / Fair
- ○ High
- ○ Very High
- ○ Not applicable to me (Already committed to non-psychiatry residency)

---

9 AFTER attending this project or session:

- ○ Very Low
- ○ Low
- ○ Neutral / Fair
- ○ High
- ○ Very High
- ○ Not applicable to me (Already committed to non-psychiatry residency)

---

10 Confidence in my CV for applying to a psychiatry residency BEFORE attending this project or session:

- ○ Very Low
- ○ Low
- ○ Neutral / Fair
- ○ High
- ○ Very High
- ○ Not applicable to me (Already committed to non-psychiatry residency)

---

11 AFTER attending this project or session:

- ○ Very Low
- ○ Low
- ○ Neutral / Fair
- ○ High
- ○ Very High
- ○ Not applicable to me (Already committed to non-psychiatry residency)

---

12 Should the MSR liaison program be implemented in other departments and/or institutions?

- ○ Strongly Disagree
- ○ Disagree
- ○ Neutral
- ○ Agree
- ○ Strongly agree

---

13 How did you hear about the MSR liaison program?

- ○ Verbal (from another student)
- ○ Verbal (from resident/faculty)
- ○ Email
- ○ Project
- ○ Other ____

---

14 If you have suggestions on how to improve further, please let us know below:

____

End of Block: Default Question Block
