# Supplementary material for: Evaluation of a Pilot Medical Student-Resident Liaison Program in Psychiatry
Source: Med Sci Educ. 2025 May 5;35(4):2021–31. doi: 10.1007/s40670-025-02404-w (PMC12532957; doi:10.1007/s40670-025-02404-w)
Supplement: Supplementary file 2 — Supplementary file2 (PDF 219 KB) [file 40670_2025_2404_MOESM2_ESM.pdf]

Introduction

Thank you all for joining today's focus group. We're here to hear about your experiences with the MSRL program. What it meant to you, how it impacted your path, and what we can learn from your insights. There are no right or wrong answers; we're interested in your honest reflections. Your feedback will help us better understand the strengths and areas for improvement in the program.

Before we begin, here are a few ground rules to help us create a respectful and open space:

Ground Rules

1. **Respect each other's perspectives** – Everyone's experience is valid. Please let others finish speaking before jumping in.
2. **Speak one at a time** – This helps with clarity and ensures everyone is heard.
3. **Confidentiality** – What's shared here stays here. Please don't share personal stories or comments outside this group.
4. **Be mindful of time** – We want to make sure everyone gets a chance to speak, so please keep comments concise when possible.
5. **No phones or distractions** – Kindly silence your phones and avoid multitasking so we can stay present in the conversation.
6. **Participation is voluntary** – You're welcome to skip any question or pass if you don't feel comfortable sharing.

Focus Group Interview Guide – MSRL Program

1. Motivation & Expectations

- What motivated you to participate in the MSRL program?
- What were your expectations going into the program?
- Looking back, do you feel your expectations were met? Why or why not?

2. Experience & Program Evolution

- Can you summarize your overall experience with the program?
- How did your experience evolve over time? Did anything shift in your understanding or engagement as the program progressed?

3. Impact on Perception of Psychiatry

- Do you think participating in the MSRL program changed your perception of psychiatry?
- If so, in what ways? If not, why do you think that is?

4. Career Confidence & CV Development

- Do you feel the program helped boost your confidence when it comes to your CV or residency application?
- In what specific ways did it influence your confidence or interest in applying to psychiatry?

5. Curricular Implications

- Now that you've completed a year in the program, do you think a similar initiative could be valuable if integrated into the medical school curriculum?
- If so, why? What aspects would be helpful to students or to other departments?
- If not, what concerns or limitations do you see?

6. Feedback & Suggestions

- If you were the program coordinator, what would you do differently?
- Are there any changes you would suggest to improve the program?
